# Stochasticity and the Molecular Mechanisms of Induced Pluripotency

**DOI:** 10.1371/journal.pone.0003086

**Published:** 2008-08-28

**Authors:** Ben D. MacArthur, Colin P. Please, Richard O. C. Oreffo

**Affiliations:** 1 Bone and Joint Research Group, Centre for Human Development, Stem Cells and Regeneration, Developmental Origins of Health and Disease, Institute of Developmental Sciences, University of Southampton, Southampton, United Kingdom; 2 School of Mathematics, University of Southampton, Southampton, United Kingdom; University of Nottingham, United Kingdom

## Abstract

The generation of induced pluripotent stem cells from adult somatic cells by ectopic expression of key transcription factors holds significant medical promise. However, current techniques for inducing pluripotency rely on viral infection and are therefore not, at present, viable within a clinical setting. Thus, there is now a need to better understand the molecular basis of stem cell pluripotency and lineage specification in order to investigate alternative methods to induce pluripotency for clinical application. However, the complexity of the underlying molecular circuitry makes this a conceptually difficult task. In order to address these issues, we considered a computational model of transcriptional control of cell fate specification. The model comprises two mutually interacting sub-circuits: a central pluripotency circuit consisting of interactions between stem-cell specific transcription factors *OCT4*, *SOX2* and *NANOG* coupled to a differentiation circuit consisting of interactions between lineage-specifying master genes.

The molecular switches which arise from feedback loops within these circuits give rise to a well-defined sequence of successive gene restrictions corresponding to a controlled differentiation cascade in response to environmental stimuli. Furthermore, we found that this differentiation cascade is strongly unidirectional: once silenced, core transcription factors cannot easily be reactivated. In the context of induced pluripotency, this indicates that differentiated cells are robustly resistant to reprogramming to a more primitive state. However, our model suggests that under certain circumstances, amplification of low-level fluctuations in transcriptional status (transcriptional “noise”) may be sufficient to trigger reactivation of the core pluripotency switch and reprogramming to a pluripotent state. This interpretation offers an explanation of a number of experimental observations concerning the molecular mechanisms of cellular reprogramming by defined factors and suggests a role for stochasticity in reprogramming of somatic cells to pluripotency.

## Introduction

Stem cells are present during all phases of development, from the embryo to the adult, and are characterized by their ability to self-renew indefinitely and differentiate along a variety of distinct lineages. Embryonic stem (ES) cells, which are derived from the inner cell mass of the developing mammalian blastocyst, are pluripotent: they have the ability to generate all embryonic tissues. In contrast, adult stem cells, which reside in small numbers in almost all adult tissues, are generally multipotent: their regenerative potential is tissue or germ-layer specific [1]–[3]. Nevertheless, since adult stem cells have the capacity to initiate specific *de novo* tissue regeneration subsequent to disease or trauma and may be derived by biopsy with reduced ethical controversy, they are of considerable current clinical and biological research interest.

The traditional conceptual model of cellular differentiation is discrete and hierarchical in nature [4]. In this view, cells can be one of a number of qualitatively different *types* (stem, progenitor or terminal cell for instance) and differentiation proceeds through a well-defined hierarchy of increasingly committed progenitor cells which act as transit populations between the most pluripotent stem cells and terminally differentiated cells, and serve to facilitate rapid clonal expansion. This process was traditionally thought to progress through a series of irreversible gene restrictions which limited dedifferentiation to more primitive states. However, recent experimental evidence suggests that under certain circumstances “terminally” differentiated cells may retain the capacity to dedifferentiate to more primitive states and possibly trans-differentiate to alternative terminal states [3], [5]–[8], although the molecular mechanisms by which this reprogramming occurs remain contentious [9].

This flexibility in the differentiation hierarchy is commonly known as lineage plasticity [3] and is perhaps most dramatically demonstrated by molecular reprogramming of adult somatic cells into so-called induced pluripotent stem (iPS) cells [10], which express the genetic and phenotypic characteristics of pluripotent ES cells. Since iPS cells potentially provide a patient-specific source of pluripotent stem cells they possess significant clinical potential [11]. Furthermore, since they are derived from adult somatic cells which are easily harvested through biopsy, the generation and clinical use of iPS cells is not associated with the same ethical controversies as human ES cells, although they are associated with significant alternative ethical issues [12]. However, current techniques to generate iPS cells rely on viral transfection of key transcription factors–a process which currently carries the inherent risk of insertional genetic mutations–and thus are unsuitable for use in a clinical setting. The search for alternative non-viral methods to generate iPS cells has focused research attention on the molecular basis of pluripotency and lineage plasticity.

Although pluripotency is controlled by multiple factors [13], in recent years the homeodomain transcription factors *OCT4* and *NANOG* and the HMG-box transcription factor *SOX2* have emerged as playing central roles in the maintenance of ES cell identity both in mice and humans [14]–[20]. For example, Boyer and co-workers used chromatin immunoprecipitation and genome-scale DNA microarrays to explore the core transcriptional circuitry in human ES cells [15], while Loh and co-workers used similar methods to identify *OCT4*, and *NANOG* targets in mouse ES cells [18]. Taken together, three key findings emerge from these studies: (1) the architecture of the core *OCT4*/*SOX2*/*NANOG* pluripotency circuit is essentially conserved between mice and humans [17]. (2) These three factors interact with each other in a coordinated manner to form a tightly regulated pluripotency circuit. In particular, the *OCT4* and *SOX2* proteins form a heterodimer which positively regulates the expression of all three of these core transcription factors [18] as well as other targets [21], [22]. Similarly *NANOG* also positively regulates expression of all three pluripotency factors [18]. Since the binding loci of these genes are often almost identical [13] and multi-protein complexes containing *OCT4* and *NANOG* may be produced by iterative immunoprecipitation in ES cells [23], evidence suggests that these three factors most-likely regulate gene expression in a cooperative manner, as part of multi-protein complexes [13]. (3) *OCT4*, *SOX2* and *NANOG* co-occupy a large set of developmentally significant target genes. In particular, they repress expression of genes associated with cellular differentiation and lineage commitment while activating expression of genes associated with self-renewal and pluripotency, including key transcription factors and members of the TGF-β and *Wnt* signaling pathways [15], [18]. As with their regulation of each other, it is likely that they also often regulate these downstream target genes in a coordinated manner [17]. Thus, cooperative interactions between *OCT4*, *SOX2* and *NANOG* are central to maintenance of the ES cell identity: appropriate expression of these 3 factors holds the cell in a pluripotent self-renewing state by activating ES cell-specific pluripotency genes and suppressing differentiation genes; while loss of expression results in up-regulation of differentiation genes, and loss of the pluripotent stem cell identity.

Commensurate with their roles in maintenance of the ES cell state, *OCT4* and *SOX2* also appear to play a central role in inducing pluripotency in somatic cells [24]. In a series of break-through papers Takahashi, Yamanaka and co-workers found that retroviral infection with just four factors–*OCT4*, *SOX2*, *KLF4* and *c-MYC*–was sufficient to transform both adult mouse and adult human fibroblastic cells to a pluripotent ES cell-like state [10], [25]. In particular, they found that subsequent to transfection, drug selection for expression of the *OCT4* target *Fbx15* [26] isolated a sub-population of cells which possessed many ES cell characteristics including the same gross morphology, the ability to form teratomas subsequent to subcutaneous injection in nude mice and the ability to differentiate along all 3 germ layers *in vitro*. However, they also found that these cells differed from ES cells in a number of crucial respects. In particular, they were unable to generate live chimeras and they differed from ES cells both in genetic and epigenetic profiles, indicating that these “first-generation” *Fbx15*-iPS cells are similar, but not identical to, ES cells.

Since these initial reports, the induction process has been refined by a number of groups [27]–[38]. In particular, iPS cells have been generated from alternative cell types including adult hepatocytes, gastric epithelial cells [31], and mesenchymal cells [30]; iPS cells have been generated without transfection with the proto-oncogene *c-MYC* [34], [36], [38]; the drug-selection process–which requires insertion of a drug-resistance gene into endogenous loci, and thus carries the risk of insertional mutations–has been replaced with selection on morphological criteria [33]; alternative combinations of key transcription factors which are sufficient to induce pluripotency have been identified [38]; and the efficiency of the reprogramming process has been improved with the use of small molecules such as DNA and histone methyltransferase inhibitors [28], [29] and histone deacetylase inhibitors [27]. Crucially, selection for *OCT4* or *NANOG* expression rather than *Fbx15* has been found to yield a more completely reprogrammed state [32], [33], [35], [37]. The resulting iPS cells are characteristically ES-like in morphology, proliferative properties, global genetic and epigenetic status [32], and in their response to key factors such as retinoic acid and leukemia inhibitory factor [35]. Additionally not only do they have the capacity to form teratomas *in vivo* [35], [37], they also support generation of viable live chimeras [35], [37] and late gestation “all iPS cell” embryos through tetraploid complementation [37]. These results currently suggest that *OCT4*- and *NANOG*-iPS cells are functionally indistinguishable from ES cells.

Taken together these reports also highlight a number of key observations which any explanation of the reprogramming process should address [17]. (1) *OCT4* and *SOX2* appear to be essential to the reprogramming process, but additional factors such as *c-MYC*, *KLF4*, *LIN28* appear to only to improve efficiency [24], [34], [36], [38]. Furthermore, *NANOG*, although a core determinant of the ES cell identity, also appears to be dispensable [10], [24], [25], [38]. (2) Viral gene expression is needed to induce pluripotency, however maintenance of the undifferentiated state is not reliant on continued transgene expression, but rather is maintained by endogenous gene expression [32], [33], [35], [37]. (3) Epigenetic reprogramming is important in establishing and maintaining the induced pluripotent state [24], [27], [29].

In this paper we shall use a computational model of stem cell differentiation to explore the molecular basis of cell fate specification and reprogramming by defined factors.

## Methods

The molecular mechanisms which underpin cell fate specification are inherently complex and difficult to interpret using experiment and intuition alone. Consequently, many authors have considered computational models of various aspects of cellular differentiation (see [39]–[51] for instance). For example, within a theoretical context it has long been suggested that distinct cell types may correspond to attractors of (generally high dimensional) genetic regulatory networks [46], [52]; an idea which has had some recent experimental validation in mammalian cells [53], [54]. Since many cell types naturally coexist in the body, the notion of a cell type as an attractor implies that the underlying dynamical regulatory system possess co-existing attractors. Consequently, differentiation has been extensively interpreted in terms of switching between co-existing attractors [55], [56] and regulatory architectures which give rise to multistability have been discussed at length (see [55] and [57], [58] and the references therein). Experimental evidence for switching between multiple discrete states during mammalian cell differentiation has also recently been provided [59], [60].

In order to better understand stem cell differentiation and reprogramming we constructed a computational model of transcriptional control of stem cell differentiation which uses these ideas and builds upon this literature. As an exemplar system we considered differentiation along the principle stromal lineages, although the regulatory architecture we shall describe occurs in a similar form in other contexts. Since mesenchymal cells have been reprogrammed to a pluripotent state [30] this represents a relevant example.

**Figure 1:**
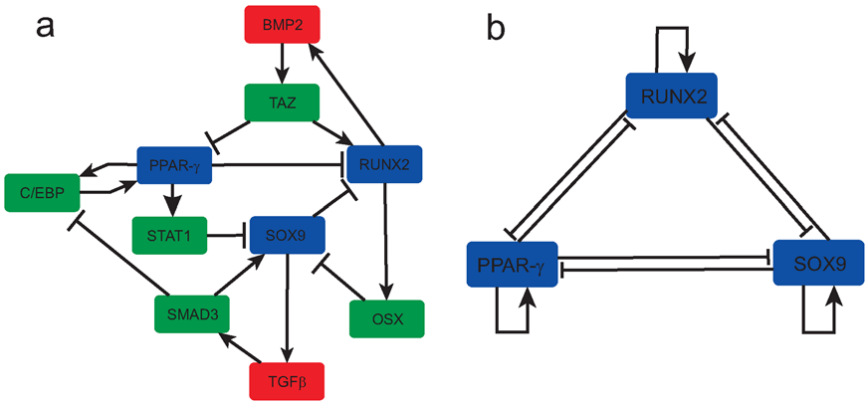
The mesenchymal transcriptional web (a) and its coarse-graining (b). Arrows indicate up-regulation, bars indicate down-regulation.

In addition to the core *OCT4/SOX2/NANOG* pluripotency circuit discussed in the introduction we also constructed an extended regulatory network for differentiation along the osteogenic (bone), chondrogenic (cartilage) and adipogenic (fat) lineages, based upon data obtained from an extensive literature search. The network we obtained consists of direct and indirect interactions between *RUNX2*, *SOX9* and *PPAR*-γ, the core lineage-specifying master genes (LSMGs) for osteogenesis, chondrogenesis, and adipogenesis respectively. The architecture of this extended network is given in Fig. 1a. In order to identify dominant interactions between the core LSMGs we considered a simple coarse-graining which emerges naturally from this extended network (see Fig. 1b). In particular, each of the core LSMGs auto-activates its own production while cross-repressing expression of the other two, either directly or through molecular intermediaries.

So, for example, BMP2 up-regulates expression of *RUNX2* while simultaneously down-regulating *PPAR*-γ through activation of the intermediary transcription factor *TAZ* [61], [62]. Additionally, *RUNX2* expression increases sensitivity to exogenous BMP2, for example via regulation of BMP receptors or SMAD signaling [63]. Thus *RUNX2* auto-activates its own expression via a positive feedback loop with BMP2 and *TAZ*. Conversely, *PPAR*-γ while a potent activator of adipogenesis also strongly inhibits osteogenesis [64], [65] by both direct suppression of *RUNX2* expression and by altering the potential of *RUNX2* to activate downstream osteogenic products [66], [67]. In contrast, *PPAR*-γ and the CCAAT/enhancer binding protein *C/EBP*-α positively regulate each others expression [68]; thus *PPAR*-γ, like *RUNX2*, also indirectly positively regulates its own production, this time through a positive feedback loop with *C/EBP*-α.

Although such a coarse-graining naturally excludes effects such as time-delays produced by indirect feedback loops, it allows us to study interactions between core transcription factors in a biologically and mathematically transparent manner. Full details of the extended regulatory network for differentiation along these stromal lineages, and its coarse-graining may be found in the supplementary materials (Text S1). We note here that the mutual cross-inhibition which emerges from this coarse-graining is also seen in other examples of cell fate specification including haematopoiesis [69] and specification of neuronal subtypes [70]. Additionally, auto-activation of lineage-determining genes is also common (for example, in the context of haematopoiesis it has been found that the myeloid determinant *PU.1* and the erythroid determinant *GATA1* are both directly auto-stimulatory [71]). Thus, the coarse-grained logic we consider (auto-activation and mutual cross-repression) is not specific to differentiation along the stromal lineages, but rather may represent a widespread form of transcriptional regulation of cell fate specification.

**Figure 2:**
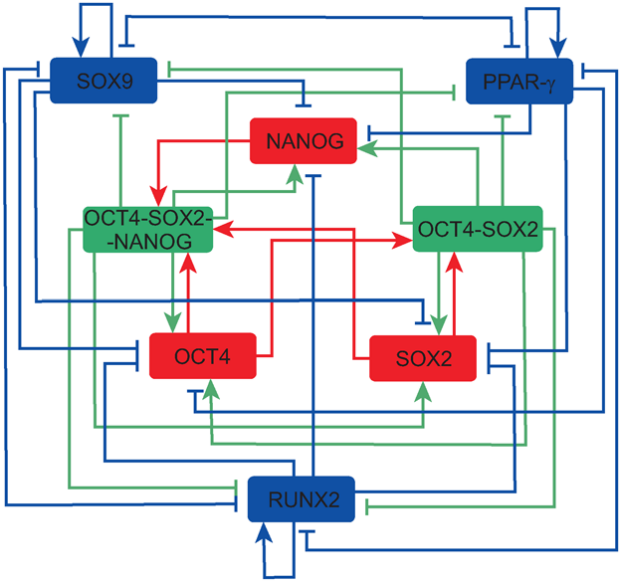
The core transcriptional circuitry for stem cell differentiation along the stromal lineages. Arrows indicate up-regulation, bars indicate down-regulation.

Molecular interactions between the core pluripotency factors (*OCT4*, *SOX2* and *NANOG*) and the stromal lineage-specifying master genes (*RUNX2*, *SOX9* and *PPAR*-γ) remain to be fully characterized. Therefore in order to connect the lineage-specifying circuit to the core pluripotency circuit, we reasoned that since *RUNX2*, *SOX9* and *PPAR*-γ are master-genes associated with differentiation, they are suppressed by *OCT4*, *SOX2* and *NANOG*, most likely through intermediate products [15], [18] and similarly that *RUNX2*, *SOX9* and *PPAR*-γ all suppress *OCT4*, *SOX2* and *NANOG*, most likely through downstream secreted growth factors. The resulting core transcriptional circuitry for differentiation from pluripotent ES cell along the principle stromal lineages is given in Fig. 2.

In order to study the dynamic behavior of this integrated network, we developed a computational model which accounts for the biological processes of interest. The model consists of a set of six coupled differential equations. For simplicity we present the model using an indexed notation. Hence, [*P_i_*] gives the nuclear concentration of the product of the *i*th pluripotency gene (PG) where *P*_1_ = *OCT4*, *P*_2_ = *SOX2* and *P*_3_ = *NANOG*. Similarly, [*L_i_*] gives the nuclear concentration of the *i*th lineage-specifying factor, where *L*_1_ = *RUNX2*, *L*_2_ = *SOX9* and *L*_3_ = *PPAR*-γ. In order to model the effect of the extracellular environment on cell fate specification, we also accounted for the effects of various exogenous stimuli on this core circuit. Hence, *s_i_* represents a combination of growth factors which stimulates differentiation along the *i*th lineage. So, for example, murine ES cells are stimulated to osteogenesis through up-regulation of *RUNX2* by retinoic acid (RA)+BMP4; to chondrogenesis through up-regulation of *SOX9* by RA+TGF-β; and to adipogenesis through up-regulation of *PPAR*-γ RA+Insulin [72]. Thus, we may consider *s*_1_ = [RA+BMP4], *s*_2_ = [RA+TGF-β] and *s*_3_ = [RA+Insulin]. Note that although these three stimuli are lineage-specific, they all contain a common element (RA), thus they all *partially* stimulate all three lineages. Furthermore, since RA suppresses *OCT4*, *SOX2* and *NANOG* expression [73], all three differentiation stimuli also suppress the core pluripotency circuit.

Based upon the logic above, on the structure of Fig. 2 and subsequent to biologically realistic simplifying assumptions (see supplementary Text S1) we obtained the following system of equations.
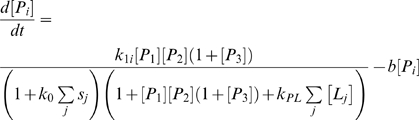
(1)

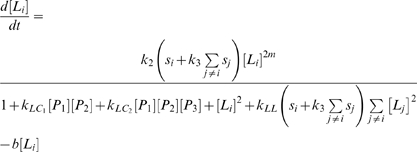
(2)Full details of the derivation of this model and a biological interpretation of the model parameters can be found in the supplementary materials (Text S1). A model of the core *OCT4*/*SOX2*/*NANOG* pluripotency circuit based upon similar assumptions has been considered elsewhere [40].

We may summarize the transcriptional logic given in Fig. 2 and Eqns. (1–2) as follows: the transcription of each core PG is activated by *OCT4*-*SOX2* and *OCT4*-*SOX2*-*NANOG* multi-protein complexes [40]. Conversely, the transcription of each LSMG is suppressed by the *OCT4*-*SOX2* and *OCT4*-*SOX2*-*NANOG* multi-protein complexes. Additionally, each LSMG auto-activates its own production, while cross-repressing that of the other LSMGs and the core PGs. Furthermore, each LSMG is up-regulated by environmental (specific and non-specific) differentiation stimuli; while each PG is down-regulated by environmental differentiation stimuli.

## Results

### Differentiation: An Irreversible Sequence of Controlled Gene Restrictions

We have conducted an extensive numerical investigation of Eqns. (1–2) and a full mathematical analysis of a simplified system (see below and supplementary materials Text S1). Here we summarize the key findings of these analyses, and discuss their biological implications. We shall focus on stable steady-state (equilibrium) solutions of this computational model. Since numerical simulations suggest that this model does not exhibit limit cycles or chaotic trajectories such equilibria represent the primary behavior of the system after any brief transients.

In accordance with the notion of a cell type as an attractor [46], [52] we associate stable equilibria with fixed cellular phenotypes. In common with other models of cellular differentiation, the system given by Eqns. (1–2) allows multiple stable steady-states (cell types) to coexist in the same environment. This multistability derives from the positive feedback loops in the transcriptional circuitry [55] and results in a sequence of genetic “switches” (that is, binary responses to continuously graded stimulus), which correspond to a sequence of successive gene restrictions.

In order to consider the molecular basis of differentiation from a pluripotent state to a terminal state, we began by considering the response of a cell initially in a pluripotent state to increasing environmental differentiation stimulus. For simplicity we consider a pluripotent ES cell in culture in which osteogenesis is initiated by addition of *s*_1_ = RA+BMP4 to the culture media in increasing doses. Since the equations have the same essential form for each lineage, our conclusions also hold quantitatively for chondrogenic induction by RA+TGF-β and adipogenic induction by RA+Insulin.

In the pluripotent state all three PGs are active (in Boolean terms, they are on) and all three LSMGs are inactive (in Boolean terms, they are off): numerical simulations suggest that this state is stable as long as the environmental stimulus (in this case the concentration of RA+BMP4) is not too high. However, as the environmental stimulus is increased the PGs are increasingly suppressed, and their expression level gradually falls. This continues until a threshold is reached, at which point the PGs can no longer maintain suppression of the LSMGs any further and one or more of the LSMGs switch on.

**Figure 3:**
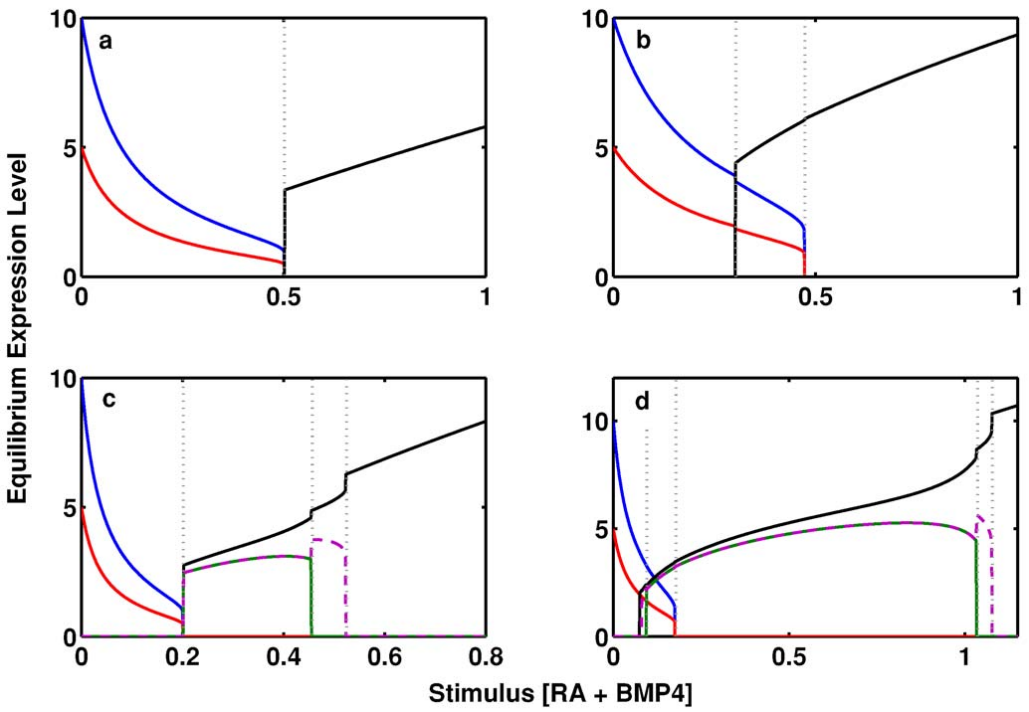
Differentiation from pluripotent stem cell to terminal osteoblast occurs either directly or through a hierarchy of increasingly committed cell types. In all panels blue indicates equilibrium *OCT4*/*SOX2* expression; red indicates equilibrium *NANOG* expression; green indicates equilibrium *SOX9* expression; dashed purple indicates equilibrium *PPAR-γ* expression; and black indicates equilibrium *RUNX2* expression. The vertical grey dotted lines mark the points when a differentiation event occurs. These figures should be read left to right since they illustrate stimulus increasing with time. (a) Differentiation straight from a pluripotent state to a terminal ostoblastic state (model parameter values: *k*_0_ = 10, *k*_11_ = 1, *k*_12_ = 0.5, *k*_2_ = 1, *k*_3_ = 0.9, *k_PL_* = 2.5, 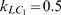, 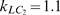, *k_LL_* = 1, *m* = 0.875, *b* = 0.1); (b) Differentiation straight from a pluripotent state to a terminal ostoblastic state via a primed state (model parameter values: *k*_0_ = 5, *k*_11_ = 1, *k*_12_ = 0.5, *k*_2_ = 5, *k*_3_ = 0.9, *k_PL_* = 0.5, 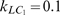, 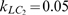, *k_LL_* = 1, *m* = 0.625, *b* = 0.1); (c) Differentiation from a pluripotent state to a terminal osteoblastic state through a hierarchy of increasingly committed tissue-specific progenitors (model parameter values: *k*_0_ = 25, *k*_11_ = 1, *k*_12_ = 0.5, *k*_2_ = 3, *k*_3_ = 0.9, *k_PL_* = 10, 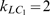, 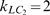, *k_LL_* = 1, *m* = 0.75, *b* = 0.1); (d) Differentiation from a pluripotent state to a terminal osteoblastic through a hierarchy of increasingly committed tissue-specific progenitors via a primed state (model parameter values: *k*_0_ = 20, *k*_11_ = 1, *k*_12_ = 0.5, *k*_2_ = 7, *k*_3_ = 0.9, *k_PL_* = 0.1, 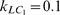, 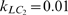, *k_LL_* = 0.75, *m* = 0.575, *b* = 0.1). Video versions of these bifurcation diagrams are given in the supplementary materials (Videos S1, S2, S3, S4). Details of the biological meaning of each of the model parameters are given in the supplementary materials (Text S1).

Numerical simulations suggest that at this point the system can display a number of different behaviors, depending upon the parameter regime. However, in all cases, we found that differentiation proceeds through a sequence of irreversible switches, corresponding to irreversible gene restrictions. Similar irreversible switches due to positive feedback loops have been observed both theoretically and experimentally in other developmental contexts (for an excellent example which combines both experiments and modeling see [74]). Fig. 3 shows examples of the various gene restriction sequences which this model exhibits for different parameter regimes. We have also included video animations of these bifurcation diagrams in the supplementary materials (Videos S1, S2, S3, S4), which present these switching dynamics in a more intuitive manner.

For some parameter values we found that the cell differentiates directly from a pluripotent state to a terminal osteoblastic state in which *RUNX2* alone is active (see Fig. 3a); while for other parameter values we found that differentiation proceeds through a sequence of intermediary states in which competing LSMGs are co-expressed before the terminal state is obtained (see Fig. 3c,d). Promiscuous expression of competing master genes has been discussed previously and associated with developmental multipotency prior to lineage commitment [75]. For example, concurrent expression of *RUNX2*, *SOX9* and *PPAR*-γ has been observed in the early stages of murine ES cell differentiation along the osteogenic lineage and associated with osteogenic/chondrogenic/adipogenic multipotency [76]. Similarly, co-expression of GATA1 and PU.1 has also been observed during haematopoiesis and similarly associated with multipotency [45]. In accordance with these observations, we associated the state in which all three LSMGs are concurrently expressed (but the PGs are off) with a *tripotent* state, and the state in which all two of the three LSMGs are concurrently expressed (but the PGs are off) with a *bipotent* state. In both the tripotent and bipotent states, pluripotency has been lost but some limited tissue-specific regenerative potential is retained.

For some parameter regimes, we found that the system can also adopt a state in which the PGs and LSMGs are co-expressed at a low level. Of all the switches we observed, only the switch from the pluripotent state to this PG/LSMG co-expressing state, was reversible: removal of stimulus from a cell in this state results in immediate transition back to the pluripotent state. Thus, we interpret the PG/LSMG co-expressing state as corresponding to a *primed* state in which the cell is preparing to commit to differentiation but commitment to a differentiating state has not yet been made. Such primed states, in which multiple LSMGs are co-expressed, have been suggested as a characteristic feature of tissue-specific (haematopoietic) stem cells [50], [77], [78]; similarly, evidence that key genes may be primed for activation in ES cells has also been presented [15], [79].

**Figure 4:**
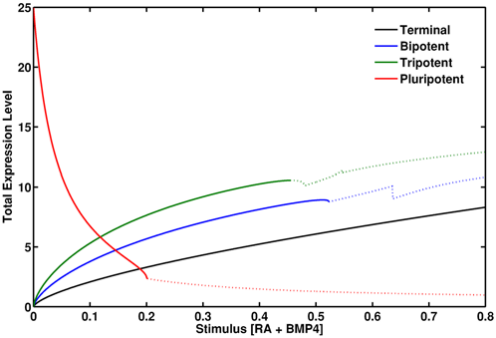
Cell types lose stability sequentially in order of developmental potency. The y-axis denotes total equilibrium gene expression ([*P*_1_]+[*P*_2_]+[*P*_3_]+[*L*_1_]+[*L*_2_]+[*L*_3_]). Bold lines indicate stable solutions; dotted lines indicate unstable solutions. Note that the cell types lose stability sequentially in order of developmental potency. Note also that for low-levels of stimuli all four cell types are concurrently stable: thus, the sequence of gene restrictions we observe are irreversible. Model parameter values are as in Fig. 3c.

In order to investigate the nature of the switches which were observed during differentiation, we examined the stability of the various cell types–pluripotent, tripotent, bipotent and terminal–as environmental stimulus is varied. Fig. 4. shows the stability of the various cell types for a biologically representative parameter regime. Importantly, for low levels of stimulus, *all* cell types are concurrently stable. As environmental stimulus is increased, the cell types lose stability sequentially in order of developmental potency. Thus, for high levels of stimulus only the terminal state is stable; while for intermediate levels of stimulus the tripotent, bipotent and terminal states are concurrently stable; and for low stimulus all four cell states are concurrently stable.

In order to investigate the nature of the switches which occur during differentiation more rigorously we also considered behavior of a related (but highly simplified) model analytically. In this simplified model the core PGs are inactive and the interactions between the LSMGs as given in Fig. 1b in response to a generic differentiation stimulus *a* are considered. Thus, this model focuses on the series of secondary bifurcations which occur after the core pluripotency circuit has been switched off. In particular, we considered the following system of equations:
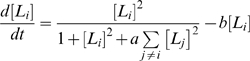
(3)Full details of our mathematical analysis of this model are provided in the supplementary materials (Text S1). A similar model of transcriptional control of differentiation is considered in [41], [42].

In supplementary Text S1 we show analytically that this simplified model supports a monotonic increasing sequence of bifurcation points, in which cell types lose stability sequentially: For 0≤*a*<*a*_1_ the multipotent state in which all three LSMGs are simultaneously expressed is stable; for 0≤*a*<*a*_2_ (where *a*_2_>*a*_1_) the bipotent states in which 2 of the LSMGs are co-expressed are stable; and for 0≤*a*<*a*_3_ (where *a*_3_>*a*_2_) the terminal states in which only one LSMG is expressed are all stable. Consequently, this simplified model exhibits multistability: for 0≤*a*<*a*_1_ all three cell types (multipotent, bipotent and terminal) are simultaneously stable. However, as stimulus is increased above the threshold *a*_1_ the multipotent state becomes unstable, gene expression is restricted and the cell is forced to adopt a bipotent state. For 0≤*a*<*a*_2_ the bipotent and terminal states are simultaneously stable but the multipotent state is unstable. However as stimulus is increased above the threshold *a*_2_ the bipotent state also becomes unstable, gene expression is further restricted and the cell is forced into a terminal state. Since the terminal states are stable for *a*<*a*_3_, removal of environmental stimulus does not result in dedifferentiation to the bipotent or multipotent states. Similarly, since *a*_2_>*a*_1_, once the cell has adopted a bipotent state it cannot dedifferentiate to a multipotent state. We believe that a similar monotonic increasing sequence of bifurcation points underpins the irreversible switching dynamics of the full model and in particular that the full system does not exhibit hysteresis within a biologically relevant parameter regime.

The central point to note from this discussion is that the molecular switches which arise from feedback loops in the core regulatory circuitry naturally give rise to a well-defined sequence of successive and irreversible gene restrictions corresponding to a directed differentiation cascade in response to specific environmental stimuli. However, the analysis we have given so far does not account for stochastic fluctuations in transcriptional status. In fact, gene expression is an inherently stochastic process [80], and transcriptional “noise” can profoundly affect cell fate decisions [81]–[86]. In the following section we explore a role for transcriptional noise in triggering stochastic transitions between co-existing attractors. In particular, we focus on exploring noise-driven transitions between the terminal state and the pluripotent state with the aim of better understanding the molecular basis of induced pluripotency.

### Cellular Reprogramming: A Role for Stochasticity

In order to consider the effect of molecular noise on system dynamics it is convenient to think of the various cell types we have identified as local minima of an energy landscape which is continually being shaped by external stimuli, and the behavior of the cell as a particle moving through this landscape to minimize its energy [87]–[89]. In this view, the local minima corresponding to the pluripotent, multipotent and bipotent cell types become increasingly shallow as differentiation stimulus is increased, until at the critical threshold points they disappear in consecutive order. Thus, a cell initially at the minimum corresponding to the pluripotent state is forced to transition through a hierarchy of local minima as environmental stimulus is increased, until finally it arrives at the “terminal” minimum corresponding to the fully differentiated state. If, at this point, environmental stimulus is gradually removed, then the local minima corresponding to the primitive cell types reappear sequentially in reverse order. However, since the switches we have identified are irreversible, the terminal minimum does not disappear upon removal of stimulus and the cell must therefore overcome an energy barrier if it is to escape the terminal state.

Stochastic fluctuations in transcriptional status (transcriptional “noise”) may be thought of, in the first instance, by analogy to the temperature of the particle in the energy landscape. If the terminal minimum is deep and/or fluctuations in transcriptional status are small (for example, if they are suppressed by epigenetic or other means) then stochastic transitions from the terminal state to alternative (more primitive) states will be rare [90]. However, if the terminal minimum is shallow and/or fluctuations in transcriptional status are large then stochastic transitions from the terminal state to alternative states will be more common. Thus, the frequency and type of noise-driven transitions between cell states depends both upon the structure of the attractor landscape and the form and amplitude of noise in the system. The notion of noise-driven transitions between co-existing attractor states has been well explored theoretically and experimentally in model organisms (see the recent reviews [84], [85] and references therein); and has recently had some experimental demonstration in mammalian cells [54].

In the previous section we examined the behavior of a cell initially in a pluripotent state to a gradual increase in environmental stimulus and found that differentiation occurs through an irreversible sequence of gene restrictions. In this section, we examine the behavior of a cell initially in a differentiated state to removal of differentiation stimulus, and investigate a role for stochasticity in triggering transitions from a differentiated state to the pluripotent state.

Fig. 4 illustrates the stability of the various cell types for varying environmental stimulus and shows that at low levels of environmental stimulus the differentiated (osteoblastic) cell adopts a state in which *RUNX2* is expressed at a low level (and all other PGs and LSMGs are OFF); and that upon complete removal of stimulus *RUNX2* expression also tends to zero. Linear stability analysis shows that in the absence of environmental stimulus this state (the origin) is stable for all model parameter values as long as the protein decay-rate *b*>0. Furthermore, analysis of Eqns. (1–2) shows that in the absence of environmental stimulus, only the origin and the pluripotent state are stable.

In order to investigate the effect of transcriptional noise on system dynamics we included a time-dependent stochastic term in Eqns. (1–2). In vector notation we denote the state of the system by **x** = [*P*_1_, *P*_2_, *P*_3_, *L*_1_, *L*_2_, *L*_3_] and the right-hand-side of Eqns. (1–2) by *F*(**x**). Thus, we considered the following set of stochastic differential equations:
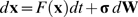
(4)Here **σ** is a diagonal matrix of constants representing the amplitude of noise, where *σ_ii_* gives the amplitude of the noise in expression of the *i*th gene, and **W** denotes a Weiner process (Brownian motion).

**Figure 5:**
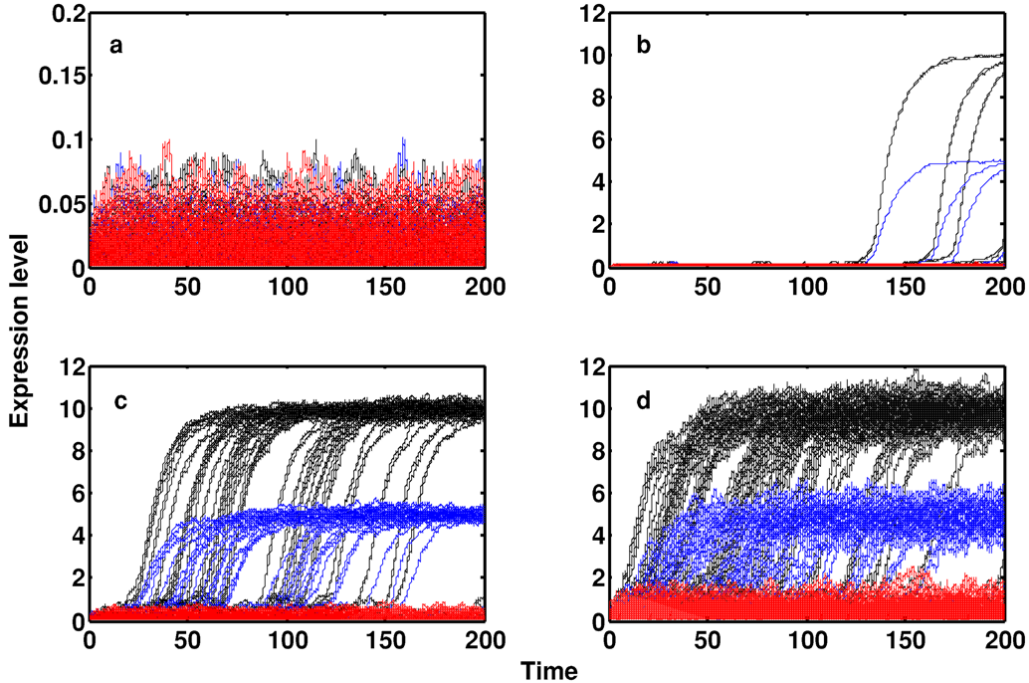
Non-specific noise can trigger reprogramming to a pluripotent state. In each panel 50 representative simulations are shown in which the expression levels of the LSMGs are given in red; the expression of *NANOG* is given in blue; and the expression levels of *OCT4* and *SOX2* are given in black. In each panel the same amplitude of noise is applied to all 6 genes (in Eqns. 4 *σ_ii_* = *σ* for all *i*). (a) *σ* = 0.01; (b) *σ* = 0.025; (c) *σ* = 0.1; (d) *σ* = 0.25. Model parameter values are as in Fig. 3c.

Fig. 5 shows some representative simulations of Eqns. 4 illustrating the behavior of a terminally differentiated osteoblast upon complete removal of differentiation stimuli (that is, starting from the origin, in the absence of environmental stimulus). In each panel 50 separate simulations are shown–each of which may be thought of as the behavior of an individual cell within an isogenic population–in which all genes are subjected to the same level of noise (*σ_ii_* = *σ* for all *i*). For low-levels of transcriptional noise stochastic transitions to the pluripotent state do not occur (Fig. 5a). However, as the level of noise in the system is increased, stochastic transitions to the pluripotent state increase in frequency (Fig. 5b–d). Finally, almost all simulations result in a transition to the pluripotent state (Fig. 5d).

**Figure 6:**
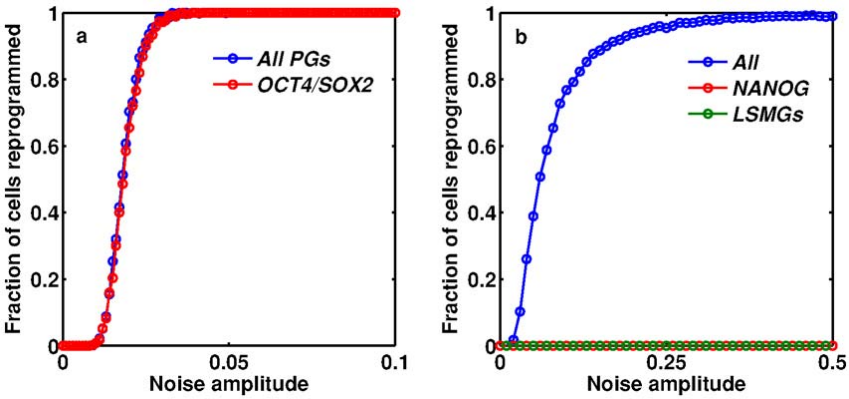
Reprogramming efficiency depends upon both the amplitude and form of transcriptional noise. (a) Noise on all PGs or *OCT4/SOX2* alone (but not the LSMGs) results in reprogramming to the pluripotent state; (b) Noise in expression of the LSMGs or *NANOG* alone does not result in reprogramming; noise in expression of all 6 genes results in reprogramming albeit less efficiently than by targeted amplification of *OCT4* and *SOX2* noise. In each case, the fraction of cells reprogrammed after 20000 time-steps is given and the results from 1000 simulations are shown, except for the case in which there is noise on all 6 genes, where the results from 5000 simulations are shown.

In order to further explore the role of stochasticity in cellular reprogramming we investigated how reprogramming efficiency varies with both the form and amplitude of transcriptional noise. Fig. 6 shows the results of these investigations. Fig 6a illustrates reprogramming efficiency (the fraction of simulations which resulted in a noise-driven transition to the pluripotent state) in the presence of noise in the expression of the PGs only (blue); and in the presence of noise in the expression of *OCT4* and *SOX2* only (red). This figure shows that only very low-level stochastic fluctuations in PG levels are needed to efficiently reprogram terminally differentiated cells to the pluripotent state. The fact that reprogramming efficiency does not change significantly when fluctuations in *NANOG* are suppressed suggests that *NANOG* is *not* required to reactivate the pluripotency switch, and therefore is not required for reprogramming. Fig. 6b illustrates reprogramming efficiency in the presence of noise in all 6 genes (blue), and in the presence of noise in the LSMGs only (green), and *NANOG* only (red). This figure shows that noise in the expression of the LSMGs or *NANOG* alone is unable to trigger stochastic transitions to the pluripotent state. However, it also illustrates that amplification of noise in the expression of all 6 genes is sufficient to trigger transitions to the pluripotent state, albeit less efficiently than by targeted amplification of *OCT4* and *SOX2* noise (compare Figs. 6a–b). This reduction in efficiency occurs since fluctuations in the PGs and the LSMGs antagonize each other: fluctuations in *OCT4/SOX2* expression serve to activate the pluripotency switch while fluctuations in the LSMGs serve to suppress the pluripotency switch.

Taken together Figs. 5–6 illustrate 2 phenomena. (1) in the absence of environmental stimulus to differentiate (for example in the ES cell culture conditions), *targeted* upregulation of *OCT4* and *SOX2* protein levels is necessary and sufficient to reprogram a differentiated cell to the pluripotent state. (2) In the absence of environmental stimulus to differentiate, *non-specific* widespread amplification of transcriptional noise–in this case, amplification of noise in the expression of all 6 genes–is also sufficient to trigger stochastic transitions to the pluripotent state. Thus, these simulations suggest that elevated levels of *OCT4* and *SOX2* protein levels are required for reprogramming. However, they also suggest that it is not necessary to target *OCT4* and *SOX2* specifically, but rather reprogramming may also be achieved by widespread non-specific amplification of transcriptional noise.

## Discussion

The molecular mechanisms of cell fate specification and reprogramming are clearly much more complex than accounted for in our simple mathematical model. Nevertheless, simplified models, such as the one presented, can be useful in conceptualizing the behavior of complex systems and can lead to insight not immediately available by experiment alone. With this caveat in mind, our results offer an explanation of a number of experimental observations concerning the molecular basis of differentiation and reprogramming by defined factors. In particular, the model we have presented shows that the molecular switches which arise from feedback loops within core transcriptional circuitry naturally give rise to a well-defined sequence of successive irreversible gene restrictions which correspond to a controlled differentiation cascade in response to environmental stimulus. Thus, in the absence of molecular fluctuations in transcriptional status, differentiation is unidirectional and cells are strongly resistant to reprogramming to a more primitive state. However, we found that under the right environmental circumstances, both targeted and non-specific amplification of stochastic fluctuations in transcriptional status was sufficient to trigger cellular reprogramming to a pluripotent state.

In the context of induced pluripotency, a central conclusion of this work is that in order to reprogram a terminally differentiated cell to a more primitive state it is necessary to supply it with sufficient “energy” to overcome the barrier holding it in the differentiated state. Current reprogramming techniques achieve this by inducing high levels of *OCT4* and *SOX2* expression from viral transgenes which in turn reactivate the endogenous pluripotency switch. However, our results suggest that since only low-level *transient fluctuations* in *OCT4* and *SOX2* protein levels are required, it is possible that this may be achieved through alternative, less invasive, methods. The fact that *transient* expression of the *OCT4* and *SOX2* proteins is sufficient to reestablish *sustained OCT4*, *SOX2* and *NANOG* gene expression explains the experimental observation that transient retroviral gene expression is needed to establish to reprogrammed state, yet pluripotency is maintained by sustained endogenous gene expression [32], [33], [35], [37]. Similarly, this result also explains why ectopic expression of *OCT4* and *SOX2* appear necessary for the reprogramming process but *NANOG* appears dispensable [10], [24], [25], [38].

Previous authors have observed that subsequent to early cellular differentiation core PGs such as *OCT4* undergo a robust multi-step silencing procedure beginning with transcriptional repression followed by an increase in histone H3 methylation and local heterochromatinization [17], [91]. This stable form of epigenetic silencing is important since it prevents harmful ectopic reactivation of PGs (ectopic activation of *OCT4* results in dysplasia in gastric epithelial tissues [92], for example). However, it also ensures that the low-level fluctuations in *OCT4* and *SOX2* protein levels needed for reprogramming are not easy to induce from endogenous genes. Thus, stable epigenetic silencing of key PGs such as *OCT4* and *SOX2* effectively suppresses spontaneous reprogramming by heavily silencing molecular fluctuations in the nuclear levels of these proteins.

Previous authors have hypothesized that additional factors such as *c-MYC* and *KLF4* facilitate reprogramming by modulating accessibility of the *OCT4* and *SOX2* loci [24], [25]. For example, *c-MYC* occupation is associated with genome-scale alteration of chromatin structure and histone accessibility [13], [93], possibly by stimulation of DNA replication [94] or by binding to multiple sites [24]; while *KLF4* regulates histone acetylation [24], [95]. Recent reports demonstrating the ability of small molecules such as DNA/histone methyltransferase inhibitors and histone deacetylase inhibitors to improve reprogramming efficiency [27]–[29] appear to support this view. Our results also support this hypothesis and suggest that by regulating *OCT4* and *SOX2* accessibility, additional reprogramming factors such as these increase sensitivity of the core pluripotency circuit to *OCT4* and *SOX2* protein levels thus increasing the probability of transitions to the pluripotent state.

In addition to confirming a central role for *OCT4* and *SOX2* in reprogramming by defined factors, our model also suggests that non-specific widespread amplification of transcriptional noise may aid reprogramming of somatic cells to a pluripotent state. Rather than targeted activation of the core pluripotency circuit by defined factors, this approach may be thought of as giving a non-specific “shake” to the system in order to realign it to the pluripotent ground state. Since a role for transcriptome-wide noise in defining mammalian cell fates has recently been experimentally demonstrated [54], we surmise that noise-processing mechanisms such as the proteasome [96] (which is involved in protein homeostasis and is used to target pre-initiation complexes and minimize noise in differentiation genes in ES cells [79]) and the *Wnt* signaling pathway (which has been implicated in filtering transcriptional noise [82]) may prove fruitful targets for future research into improving the efficiency of reprogramming protocols. Such approaches are currently under experimental investigation in our laboratory.

We conclude by noting that although iPS cells are not associated with the same ethical concerns as the derivation of human ES cells, they are nevertheless associated with their own set of significant ethical considerations [12]. It is important that these are addressed concurrently with research in this area.

## Supporting Information

Text S1 Transcriptional control of the mesenchymal lineages; Derivation of model equations; Mathematical details
(0.16 MB PDF)

Video S1 A video version of Fig. 3a. Green indicates gene expression, red indicates [RA+BMP4].
(1.27 MB AVI)

Video S2 A video version of Fig. 3b. Green indicates gene expression, red indicates [RA+BMP4].
(1.30 MB AVI)

Video S3 A video version of Fig. 3c. Green indicates gene expression, red indicates [RA+BMP4].
(1.04 MB AVI)

Video S4 A video version of Fig. 3d. Green indicates gene expression, red indicates [RA+BMP4].
(1.48 MB AVI)
