# Supplementary material for: Stochasticity and the Molecular Mechanisms of Induced Pluripotency
Source: PLoS One. 2008 Aug 28;3(8):e3086. doi: 10.1371/journal.pone.0003086 (PMC2517845; doi:10.1371/journal.pone.0003086)
Supplement: Text S1 — Transcriptional control of the mesenchymal lineages; Derivation of model equations; Mathematical details (0.16 MB PDF) [file pone.0003086.s001.pdf]

Stochasticity and the Molecular Mechanisms of Induced Pluripotency

Supplementary Materials

Ben D. MacArthur
Bone and Joint Research Group,
Centre for Human Development, Stem Cells and Regeneration,
Institute of Developmental Sciences,
University of Southampton, Southampton, SO16 6YD, UK

Colin P. Please
School of Mathematics, University of Southampton
Southampton, SO17 7BJ, UK

Richard O. C. Oreffo
Bone and Joint Research Group,
Centre for Human Development, Stem Cells and Regeneration,
Institute of Developmental Sciences,
University of Southampton, Southampton, SO16 6YD, UK

1 Transcriptional control of the Mesenchymal lineages

The Runt domain family member *RUNX2* (alternatively known as *CBFA1* or *OSF2*) is a well known transcriptional activator of osteogenesis [1, 2]. For example, Karsenty and co-workers established that *RUNX2* binds to the promoter region of multiple osteoblast-specific genes (including osteocalcin, collagen I, bone sialoprotein, and osteopontin), and is a key regulator of osteoblast differentiation *in vivo* [3]. In particular, they showed that *RUNX2* controls expression of osteocalcin, the only known gene to be expressed in osteoblasts and no other matrix producing cells. Analogously, the transcription factor *SOX9* has been identified as essential for cartilage formation. Bi and co-workers showed that *SOX9* binds to the promoter region of both collagen 2a1 and collagen 11a2 genes [4], both of which are essential for healthy cartilage formation. Similarly the transcription factor *PPAR*-γ is essential for adipogenesis [5, 6] binding to, for example, the adipocyte specific *AP2* enhancer [7]. Furthermore, *PPAR*-γ is a potent activator of adipogenesis in nonadipogenic cells. For example, Tontonoz and co-workers demonstrated that retroviral transfection with the *PPAR*-γ gene induces adipogenesis in cultured fibroblasts [6].

Since all three of these master genes are expressed in early mesenchymal progenitors (murine fetal derived mesenchymal stem cells coexpress *RUNX2* and *PPAR*-γ [8] but do not express downstream lineage specific markers such as osteocalcin and *AP2*; while *RUNX2* and *SOX9* are expressed in early osteochondral progenitors [9]) these results suggest that interactions between the *RUNX2/SOX9/PPAR*-γ transcriptional trio determine *dynamic* cell fate decisions in these mesenchymal lineages.

In fact, these three transcription factors are known to interact with each other either directly or via downstream proteins. For example, recent results have shown that BMP2 upregulates expression of *RUNX2* while simultaneously downregulating transcription of *PPAR*-γ through activation of the intermediary transcription factor *TAZ* [10, 11]. Additionally, it has also been suggested that *RUNX2* expression may also increase sensitivity to exogenous BMP2, for example via regulation of BMP receptors or SMAD signaling [12], thus introducing an indirect autoactivating feed-forward loop in *RUNX2* expression. Conversely, *PPAR*-γ while a potent activator of adipogenesis also strongly inhibits osteogenesis [13, 14] by both direct suppression of *RUNX2* expression and altering the potential of *RUNX2* to activate downstream osteogenic products [15, 16]. On the other hand *PPAR*-γ and the CCAAT/enhancer binding protein *C/EBP*-α positively regulate each others expression [17]. Thus *PPAR*-γ, like *RUNX2*, also indirectly positively regulates its own production.

Eames and co-workers showed that during skeletal development in chick embryos synergistic patterns of co-expression of both *SOX9* and *RUNX2* determine osteo-chondral cell fates [18]. In particular, they provide direct evidence that overexpression of *SOX9* can directly inhibit expression of *RUNX2* during skeletogenesis. Alternatively osterix, a transcriptional regulator of osteogenesis downstream of *RUNX2* [19], has been suggested as a negative regulator of *SOX9* expression [20]. Furthermore, Interleukin-6 has been shown to down-regulate *SOX9* expression

through the *STAT1/3* pathway in bovine articular chondrocytes [21], while *PPAR*-γ has been shown to upregulate *STAT1* in NIH3T3 transfected fibroblasts [22]: introducing a repression of *SOX9* expression by *PPAR*-γ through *STAT1*.

The TGF-β signaling pathway is central to control of chondrogenesis and adipogenesis via *SOX9* and *PPAR*-γ. For example, TGF-β stimulates chondrogenesis through SMAD3 upregulation of *SOX9* [23] while simultaneously inhibiting adipogenesis by repressing *C/EBP* transactivation function also via *SMAD3* [24]. However full details of how *SOX9* and *PPAR*-γ expression are balanced during chondrogenesis and adipogenesis are currently lacking. Therefore, in order to complete the network we reasoned that *SOX9* increases sensitivity to TGF-β signaling, thus introducing autoactivation of *SOX9* via TGF-β and *SMAD3* and cross-repression of *PPAR*-γ by *SOX9* via TGF-β, *SMAD3* and *C/EBP*.

Taken together these interactions form the regulatory network given in Fig. 1 of the main text. This network may be coarse-grained by observing that each master-gene either directly or indirectly autoactivates its own expression, while cross-repressing those of the other two. We note here that since positive feedback loops can be destabilizing, it is likely that alternative negative feedback mechanisms are also present to balance the autoactivation loops. So, for example, both in mice and rats *RUNX2* directly down-regulates its own promoter activity [25] indicating that there is a competition between auto-upregulation (via BMP2 and *TAZ*) and direct auto-downregulation of *RUNX2*. We anticipate that analogous negative feedback controls are present during chondrogenesis and adipogenesis and act to stabilize *SOX9* and *PPAR*-γ expression (for more details see model derivation below).

2 Derivation of Model Equations

Here we derive the computational model based upon the integrated transcriptional logic given in Fig. 2 in the main text. The core of our model is derived from the following stoichiometric equations which describe transcription factor binding to gene promoters:

$$\begin{aligned}
P_1 + P_2 &\overset{k_{C_1}}{\rightleftharpoons} C_1 \\
P_1 + P_2 + P_3 &\overset{k_{C_2}}{\rightleftharpoons} C_2 \\
C_j + F_{P_i} &\overset{K_{P_iC_j}}{\rightleftharpoons} B_{P_iC_j} \\
L_j + F_{P_i} &\overset{K_{P_iL_j}}{\rightleftharpoons} B_{P_iL_j} \\
C_j + F_{L_i} &\overset{K_{L_iC_j}}{\rightleftharpoons} B_{L_iC_j} \\
L_j + L_j + F_{L_i} &\overset{K_{L_iL_j}}{\rightleftharpoons} B_{L_iL_j}.
\end{aligned}$$

In total there are 32 stoichiometric equations, and we have used an index notation for simplicity of nomenclature: P_i for $i = 1, 2, 3$ represent the three pluripotency genes (PGs), with $P_1 = OCT4$, $P_2 = SOX2$ and $P_3 = NANOG$. Similarly, L_i, for $i = 1, 2, 3$ represent the three lineage-specifying master genes (LSMGs) with $L_1 = RUNX2$, $L_2 = SOX9$ and $L_3 = PPAR\text{-}\gamma$. We assume that *OCT4*, *SOX2* and *NANOG* proteins associate to form an *OCT4-SOX2* complex, represented by C_1, and an *OCT4-SOX2-NANOG* complex, represented by C_2. Free binding sites in the promoters of the PGs are denoted F_{P_i}, while free binding sites in the promoters of the LSMGs are denoted F_{L_i}. Bound sites in the promoters of the PGs are denoted B_{P_iY}, where $Y \in \{C_j, L_j\}$ depending on whether the site is bound by one of the pluripotency complexes or a LSMG respectively. Similarly, bound sites in the promoters of the LSMGs are denoted B_{L_iY}. The parameters k_{C_1}, k_{C_2} and K_{XY} (where $X \in \{P_i, L_i\}$ and $Y \in \{C_j, L_j\}$) are the equilibrium dissociation constants for the respective reactions. Here we have assumed that the LSMGs bind co-operatively to each others promoters with binding site affinity 2. This assumption has been taken in other computational models of transcription factor binding [26], and ensures that the resulting feedback loops are nonlinear, yet also keeps the resulting mathematics transparent. In order to determine the effect of binding site affinity on model solutions, we conducted extensive numerical simulations using a range of other binding affinities. We found that the qualitative conclusions of the model are not significantly affected by variations in transcriptional binding site affinity.

Assuming the reactions are in quasi-steady state gives the equations:

$$\begin{aligned}
C_1 &= k_{C_1} P_1 P_2 \\
C_2 &= k_{C_2} P_1 P_2 P_3 \\
B_{P_iC_j} &= K_{P_iC_j} F_{P_i} C_j \\
B_{P_iL_j} &= K_{P_iL_j} F_{P_i} L_j \\
B_{L_iC_j} &= K_{L_iC_j} F_{L_i} C_j \\
B_{L_iL_j} &= K_{L_iL_j} F_{L_i} L_j^2.
\end{aligned}$$

We can now determine T_{P_i} the total number of binding sites in the promoter regions of each of the PGs:

$$\begin{aligned}
\text{Total Sites} &= \text{Free Sites} + \text{Bound Sites} && (1) \\
T_{P_i} &= F_{P_i} + \sum_{j=1}^{2} B_{P_iC_j} + \sum_{j=1}^{3} B_{P_iL_j} && (2) \\
&= F_{P_i} + \sum_{j=1}^{2} K_{P_iC_j} F_{P_i} C_j + \sum_{j=1}^{3} K_{P_iL_j} F_{P_i} L_j, && (3) \\
&= F_{P_i} \left(1 + \sum_{j=1}^{2} K_{P_iC_j} C_j + \sum_{j=1}^{3} K_{P_iL_j} L_j \right), && (4)
\end{aligned}$$

and T_{L_i} the total number of binding sites in the promoter regions of each of the LSMGs:

$$\text{Total Sites} = \text{Free Sites} + \text{Bound Sites} \tag{5}$$

$$T_{L_i} = F_{L_i} + \sum_{j=1}^{2} B_{L_i C_j} + \sum_{j=1}^{3} B_{L_i L_j} \tag{6}$$

$$= F_{L_i} + \sum_{j=1}^{2} K_{L_i C_j} F_{L_i} C_j + \sum_{j=1}^{3} K_{L_i L_j} F_{L_i} L_j^2, \tag{7}$$

$$= F_{L_i} \left(1 + \sum_{j=1}^{2} K_{L_i C_j} C_j + \sum_{j=1}^{3} K_{L_i L_j} L_j^2 \right). \tag{8}$$

These equations may be rearranged to give the total number of free sites in the promoters of the PGs and LSMGs:

$$F_{P_i} = \frac{T_{P_i}}{1 + \sum_{j=1}^{2} K_{P_i C_j} C_j + \sum_{j=1}^{3} K_{P_i L_j} L_j}, \tag{9}$$

$$F_{L_i} = \frac{T_{L_i}}{1 + \sum_{j=1}^{2} K_{L_i C_j} C_j + \sum_{j=1}^{3} K_{L_i L_j} L_j^2}. \tag{10}$$

The extended transcriptional network we have derived suggests that competition between signaling pathways ensures that generically each master-gene regulates its own promoter in a biphasic manner: at low nuclear concentrations of transcription factor gene expression is activated upon binding; while at high nuclear concentrations, gene expression is repressed by transcriptional binding. This biphasic relationship between transcription factor concentration and gene expression ensures that autoactivating feedback loops are tightly controlled, and has been experimentally observed in mesenchymal systems. For example, the trans-acting factor protein *AP2* regulates both cartilage-derived retinoic acid-sensitive protein (*CD-RAP*) and insulin-like growth factor binding protein-5 Gene (*IGFBP5*) expression in a biphasic manner [27, 28], possibly by transcriptional self-interference in which *AP2* protein molecules interact with putative co-factors to interfere with *AP2* regulation of gene expression [29].

In order to capture the essential features of biphasic regulation in a tractable manner, we assumed that the protein product of each LSMG is inactivated by binding to putative co-factor(s), and that binding to co-factors is increased at high concentrations possibly due to co-regulation of cofactor expression or post-transcriptional modification of LSMG products. Letting $[L_{iA}]$ denote the nuclear concentration of the activating form of L_i we make the phenomenological hypothesis:

$$[L_{iA}] \propto [L_i]^m \tag{11}$$

where m is a constant in the range $0 \leq m \leq 1$. Note that this law ensures that $[L_{iA}]/[L_i] \to 0$ as $[L_i]$ saturates.

We assume that the rate of production of the protein product of each gene is proportional to the number of binding sites in its promoter bound by an activator. Thus,

$$\text{Rate of Production of } P_i \propto F_{P_i} \sum_{j=1}^{2} K_{P_i C_j} C_j \tag{12}$$

$$\text{Rate of Production of } L_i \propto K_{L_i L_i} F_{L_i} L_{iA}^2 = K_{L_i L_i} F_{L_i} L_i^{2m} \tag{13}$$

Here we have assumed that the *OCT4-SOX2* and *OCT4-SOX2-NANOG* complexes are always in an activating form. Substituting Eqs. (9-10) into the above equations, and assuming that the protein products decay at constant rates b_{P_i} and b_{L_i} we obtain the following set of nonlinear ordinary differential equations to describe the concentrations of the various species

$$\frac{d[P_i]}{dt} = \frac{A_i \sum_{j=1}^{2} K_{P_iC_j}[C_j]}{1+\sum_{j=1}^{2} K_{P_iC_j}[C_j]+\sum_{j=1}^{3} K_{P_iL_j}[L_j]} - b_{P_i}[P_i], \tag{14}$$

$$\frac{d[L_i]}{dt} = \frac{B_i K_{L_iL_i}[L_i]^{2m}}{1+\sum_{j=1}^{2} K_{L_iC_j}[C_j]+\sum_{j=1}^{3} K_{L_iL_j}[L_j]^2} - b_{L_i}[L_i]. \tag{15}$$

Here A_i and B_i are constants of proportionality which incorporate T_{P_i} and T_{L_i}. Thus we have adopted Michaelis-Menten type kinetics with generalized Hill coefficients [30].

In order to clarify analysis, we make the following biologically reasonable simplifying assumptions: (1) Binding of the *OCT4-SOX2* and *OCT4-SOX2-NANOG* complexes to the promoters of the PGs and LSMGs occurs at the same rate. Thus, we take $K_{P_iC_1} = K_{PC_1}$, $K_{P_iC_2} = K_{PC_2}$, $K_{L_iC_1} = K_{LC_1}$, and $K_{L_iC_2} = K_{LC_2}$ for all i. (2) Binding of the protein-products of all LSMGs to the promoters of the PGs occurs at the same rate for all PGs. Thus, we take $K_{P_iL_j} = K_{PL}$ for all i, j. (3) Binding of the protein product of all LSMGs to their own promoter occurs at the same rate. Thus, we take $K_{L_iL_i} = K_L$ for all i. (4) Binding of the protein product of LSMG i to the promoter of LSMG $j \neq i$ occurs at a base rate independent of i and j (we shall consider how external stimuli affect this base binding rate in more detail below). Thus, we take $K_{L_iL_j} = K^i_{LL}$ for all $i, j \neq i$. (5) The constants of proportionality relating rate of production of the protein product to base rate of activator binding to the promoter is the same for all PGs and the same for all LSMGs. Thus, we take $A_i = A$ and $B_iK_L = \bar{k}_{2i}$ for all i (we shall consider how external stimuli affect these base binding rates in more detail below). (6) All protein products decay at the same constant rate. Thus, we take b_{P_i} and $b_{L_i} = \beta$ for all i.

Additionally, we nondimensionalise by taking the following scalings:

$$t = \frac{1}{K_L^{2m-1}}\hat{t} \qquad [P_1] = \frac{1}{\sqrt{K_{PC_1}}}[\hat{P_1}] \qquad [P_2] = \frac{1}{\sqrt{K_{PC_1}}}[\hat{P_2}] \qquad [P_3] = \frac{K_{PC_1}}{K_{PC_2}}[\hat{P_3}] \qquad [L_i] = \frac{1}{\sqrt{K_L}}[\hat{L_i}]$$

where hatted variables represent nondimensional quantities.

Thus, we get

$$\frac{d[P_i]}{dt} = \frac{\bar{k}_{1i}[P_1][P_2](1+[P_3])}{1+[P_1][P_2](1+[P_3])+k_{PL}\sum_{j=1}^{3}[L_j]} - b[P_i] \tag{16}$$

$$\frac{d[L_i]}{dt} = \frac{\bar{k}_{2i}[L_i]^{2m}}{1+k_{LC_1}[P_1][P_2]+k_{LC_2}[P_1][P_2][P_3]+[L_i]^2+\bar{k}^i_{LL}\sum_{j=1\neq i}^{3}[L_j]^2} - b[L_i] \tag{17}$$

where $k_{PL} = K_{PL}/\sqrt{K_L}$, $k_{LC_1} = K_{LC_1}/K_{PC_1}$, $k_{LC_2} = K_{LC_2}/K_{PC_2}$, $\bar{k}^i_{LL} = K^i_{LL}/K_L$, $b = \beta/K_L^{2m-1}$, $\bar{k}_{11} = \bar{k}_{12} = A\sqrt{K_{PC_1}}/K_L^{2m-1}$ and $\bar{k}_{13} = AK_{PC_2}/K_{PC_1}K_L^{2m-1}$, and we have dropped hats for notational simplicity.

In order to examine cellular differentiation, we assume that the external environment contains specific elements

which stimulate cellular differentiation. So, for example, murine ES cells are stimulated to osteogenesis by upregulation of *RUNX2* by Retinoic Acid (RA) + BMP4; to chondrogenesis by upregulation of *SOX9* by RA + TGF-β; and to adipogenesis by upregulation of *PPAR*-γ by RA + Insulin [31]. Thus, we consider three specific stimuli: s_1 = [RA + BMP4], s_2 = [RA + TGF-β] and s_3 = [RA + Insulin]. Note that although these three stimuli are lineage-specific, they all contain a common element (RA), thus they all *partially* stimulate all three lineages. Similarly, since RA suppresses *OCT4*, *SOX2* and *NANOG* expression [32], the three lineage-specific stimuli all also suppress expression of the core PGs.

Thus, we assumed that the rate of binding of activators to PG promoters is inversely proportional to the external concentration of nonspecific differentiation stimuli; while the rate of binding activators to LSMG promoters is proportional to the external concentration of specific and nonspecific differentiation stimuli. Consequently, we allowed $\bar{k}_{1i}$, $\bar{k}_{2i}$ and $\bar{k}^i_{LL}$ to depend upon external stimuli and assumed that all other model parameters were constant. For the sake of simplicity, we assumed that stimulatory factors are not produced or degraded, but rather are present in the extracellular environment at a known concentration, as would be the case during an *in vitro* experiment. Specifically, we took

$$\bar{k}_{1i} = \frac{k_{1i}}{1 + k_0 \sum_j s_j}, \tag{18}$$

$$\bar{k}_{2i} = k_2(s_i + k_3 \sum_{j \neq i} s_j), \tag{19}$$

$$\bar{k}^i_{LL} = k_{LL}(s_i + k_3 \sum_{j \neq i} s_j). \tag{20}$$

Here k_0, k_{1i}, k_2, k_3 and k_{LL} are constants and s_i represents the combination of growth factors which stimulates differentiation along the ith lineage. For simplicity we have assumed that stimulation of the ith lineage by the jth stimulus occurs at the same rate for all $i \neq j$, and that the PGs are equally suppressed by all three stimuli. Substituting Eqs. (18-20) into Eqs. (16-17) gives

$$\frac{d[P_i]}{dt} = \frac{k_{1i}[P_1][P_2](1 + [P_3])}{(1 + k_0 \sum_j s_j)(1 + [P_1][P_2](1 + [P_3]) + k_{PL} \sum_j [L_j])} - b[P_i] \tag{21}$$

$$\frac{d[L_i]}{dt} = \frac{k_2(s_i + k_3 \sum_{j \neq i} s_j)[L_i]^{2m}}{1 + k_{LC_1}[P_1][P_2] + k_{LC_2}[P_1][P_2][P_3] + [L_i]^2 + k_{LL}(s_i + k_3 \sum_{j \neq i} s_j) \sum_{j \neq i}[L_j]^2} - b[L_i] \tag{22}$$

which are the equations given in the main text.

The model has the following 11 free parameters: k_{1i} for $i = 1, 2, 3$ ($k_{11} = k_{12}$) represent the relative base rates of binding of the *OCT4*-*SOX2* and *OCT4*-*SOX2*-*NANOG* complexes to the PG promoters; k_0 represents the sensitivity the PGs to exogenous inhibition with RA; K_{PL} represents the relative rate of binding of the LSMGs to the promoters of the PGs; k_2 represents the sensitivity the LSMGs to exogenous activation by lineage-specific stimulus; k_3 represents the relative sensitivity of the LSMGs to nonspecific stimulus; k_{LC_1} represents the relative rate of binding of the *OCT4*-*SOX2* complex to the promoters of the LSMGs; k_{LC_2} represents the relative rate of binding of the *OCT4*-*SOX2*-*NANOG* complex to the promoters of the LSMGs; K_{LL} represents the relative rate

of binding of LSMG i to the promoters of LSMG $j \neq i$; b represents the relative half-life of the protein products of the LSMGs and PGs; and $0 < m \leq 1$ gives the relative degree of transcriptional self-interference for each of the LSMGs and characterizes the biphasic form of the autoactivating feedback loops.

3 Mathematical Details

We include here a mathematical derivation of the existence and stability of steady-state solutions to a simplified system which captures the essential secondary bifurcation behavior of Eqs. (21)-(22). In particular, we assume that the pluripotency switch is off ($[P_i] = 0$ for all i) and consider the system:

$$\frac{dx_i}{dt} = \frac{x_i^{2m}}{1 + x_i^2 + a\sum_{j\neq i} x_j^2} - bx_i, \tag{23}$$

where a is a generic differentiation stimulus. We shall consider the general case of N distinct lineages ($i = 1 \ldots N$) and we have simplified notation by denoting $[L_i]$ by x_i. In general $0 \leq m \leq 1$. We shall discuss solutions in the limiting case $m = 1$ and indicate how this limiting case illuminates the more general solution structure.

The equations of interest are:

$$\frac{dx_i}{dt} = \frac{x_i^2}{1 + x_i^2 + a\sum_{j\neq i} x_j^2} - bx_i = f_i, \tag{24}$$

In general Eqs. (24) may possess steady-state solutions in which genes are transcribed at various unequal (but constant) rates. Let α_i be the steady-state expression level of gene i where α_i is a solution to

$$\alpha_i^2 - b\alpha_i\big[1 + \alpha_i^2 + (1-a)\alpha_i^2 + a\sum_j \alpha_j^2\big] = 0.$$

Upon rearrangement this gives $\alpha_i = 0$ or

$$\alpha_i - b(1-a)\alpha_i^2 = \alpha_j - b(1-a)\alpha_j^2 \tag{25}$$

for all $i \neq j$. Thus α_i and α_j satisfy the *same* quadratic equation. Therefore, the set of active genes can be partitioned into at most 2 distinct blocks, with transcriptional levels α and β respectively, where from Eq. (25)

$$\beta = \frac{1}{b(1-a)} - \alpha.$$

Thus generic steady-state solutions to Eqs. (24) have the form:

$$\mathbf{x}^* = (\underbrace{\alpha, \alpha, \ldots, \alpha}_{p \text{ times}}; \underbrace{\beta, \beta, \ldots, \beta}_{q \text{ times}}; \underbrace{0, 0, \ldots, 0}_{r \text{ times}}), \tag{26}$$

where $p + q + r = N$.

The Jacobian $\mathbf{J}_N(\mathbf{x}^*)$ of the system at $\mathbf{x}^*$ is the $N \times N$ matrix with block-diagonal form:

$$\mathbf{J}_N(\mathbf{x}^*) = \begin{pmatrix} \mathbf{J}_{pq} & 0 \\ 0 & \mathbf{J}_r \end{pmatrix} \tag{27}$$

where $\mathbf{J}_{pq}$ is the $(p+q) \times (p+q)$ Jacobian of the system projected onto $X \cong \mathbb{R}^{p+q}$, the subspace spanned by $\{e_i : i = 1, 2, \ldots, p+q\}$, and $\mathbf{J}_r$ is the $r \times r$ Jacobian of the system projected onto $Y \cong \mathbb{R}^r$, the subspace spanned by $\{e_i : i = p+q+1, p+q+2, \ldots, N\}$, where $\{e_i\}$ is the standard basis of $\mathbb{R}^N$. The eigenvalues of $\mathbf{J}_N(\mathbf{x}^*)$ are therefore the eigenvalues of $\mathbf{J}_{pq}$ along with those of $\mathbf{J}_r$.

Since $\mathbf{x} = \mathbf{0}$ for all $\mathbf{x} \in Y$, the Jacobian $\mathbf{J}_r$ is diagonal: $\mathbf{J}_r = \mathrm{diag}(F, F, \ldots, F)$ where

$$F = \left.\frac{\partial f_i}{\partial x_i}\right|_{x=\mathbf{x}^*} = -b. \tag{28}$$

Thus, $\mathbf{J}_r$ has only one eigenvalue: $-b$ (with multiplicity r). Since $b > 0$ the stability of the generic fixed point given in Eq. (26) is determined solely by the eigenvalues of $\mathbf{J}_{pq}$. Since X is flow invariant, this means that we need only consider solutions of the form

$$\mathbf{x} = (\underbrace{\alpha, \alpha, \ldots, \alpha}_{p \text{ times}}; \underbrace{\beta, \beta, \ldots, \beta}_{q \text{ times}}) \tag{29}$$

of the system projected onto X. Doing so reduces the dimension of the problem from N to $N - r$.

Observe that the system given by Eqs. (24) 'looks the same' if we permute the x_is. That is, it remains unchanged under the action of the symmetric group S_N permuting the co-ordinates (the system given by Eqs. (24) is S_N-equivariant). The bifurcation behaviour of S_N-equivariant systems has been considered in some depth [33, 34]. A central result of these analyses is that solution branches in which species are expressed at unequal levels (as is the case in Eq. (29)) bifurcating from homogeneous steady-state solutions (in which all species are active at the *same level*) are generically unstable. This implies that S_N-equivariant symmetry breaking bifurcations are generically 'jump' bifurcations. Our extensive numerical investigations suggest furthermore that steady-state solutions in which genes are expressed at different non-zero levels are *always* unstable. Without loss of generality, stable steady-state solutions to Eqs. (24) therefore generically have the form

$$\mathbf{x}^* = (\underbrace{\alpha, \alpha, \ldots, \alpha}_{p \text{ times}}; \underbrace{0, 0, \ldots, 0}_{r \text{ times}}) \tag{30}$$

where $p + r = N$.

Notice that the Jacobian at $\mathbf{x}^*$ still has the diagonal form given in Eq. (27) with $q \equiv 0$. Thus the stability of all remaining steady-state solutions in the N dimensional system can be determined by consideration of homogeneous all-ON solutions projected onto X for varying dimension p. In other words, once a gene has been switched OFF we can (as far as stability calculations are concerned) consider it removed from the system, and examine the behavior of the system in $N - 1$ dimensions.

Consider now the general case in which p genes are expressed at the same level. Steady-state solutions then have the form $\mathbf{x}^* = (\alpha, \alpha \ldots, \alpha)$, where α is a solution to:

$$\alpha - b\big[1 + \alpha^2 + a(p-1)\alpha^2\big] = 0. \tag{31}$$

Thus,

$$\alpha = \frac{1 \pm \sqrt{(1 - 4b^2[a(p-1)+1])}}{2b[a(p-1)+1]}. \tag{32}$$

For $p \geq 2$, both branches of Eq. (32) are non-negative and real when

$$a \leq \frac{1 - 4b^2}{4b^2(p-1)}.$$

Since $a > 0$, we also require that $b \leq \frac{1}{2}$ for existence. For $p = 1$, we require only $b \leq \frac{1}{2}$ for existence.

We turn our attention now to the stability of the homogeneous steady-state solution $\mathbf{x}^*$ which is (linearly) stable if $\mathbf{Re}(\lambda) < 0$ for all eigenvalues λ of the Jacobian $\mathbf{J}_p(\mathbf{x}^*)$. Since matrix representation of elements of the symmetric group S_p commute with the Jacobian of the system projected onto X, the steady-state $\mathbf{x}^* = (\alpha, \alpha, \ldots, \alpha)$ has only 2 distinct eigenvalues: λ_1 and λ_2,

$$\lambda_1 = F - (p-1)G, \tag{33}$$

$$\lambda_2 = F - G, \tag{34}$$

where λ_2 has multiplicity $p - 1$, and

$$F = \left.\frac{\partial f_i}{\partial x_i}\right|_{\mathbf{x}=\mathbf{x}^*} \text{ and } G = \left.\frac{\partial f_i}{\partial x_j}\right|_{\mathbf{x}=\mathbf{x}^*}.$$

Upon differentiating and simplifying using Eq. (31) we get:

$$\lambda_1 = b(1 - 2\alpha b) - 2ab^2\alpha(p-1), \tag{35}$$

$$\lambda_2 = b(1 - 2\alpha b) + 2ab^2\alpha. \tag{36}$$

For a, b, α and p positive, $\lambda_1 < \lambda_2$ so $\mathbf{x}^*$ is stable for $\lambda_2 < 0$. Thus, both branches of solutions in Eq. (32) are unstable for $a \geq 1$.

For $a < 1$, $\mathbf{x}^*$ is stable when

$$\alpha > \frac{1}{2b(1-a)}. \tag{37}$$

Substituting the lower branch of solutions from Eq. (32) into Eq. (37) and rearranging gives stability for the lower branch when

$$\sqrt{1 - 4b^2\big[a(p-1)+1\big]} < \frac{-ap}{1-a}.$$

So the lower branch of solutions is unstable for *all* real positive a. Similarly, the upper branch of solutions is stable for

$$\sqrt{1 - 4b^2\big[a(p-1)+1\big]} > \frac{ap}{1-a},$$

with bifurcation occurring at equality, when

$$a = a_p = 1 - \frac{1}{8b^2}\big(p + 1 \pm \sqrt{(p+1)^2 - 16b^2 p}\big).$$

The positive root of this equation is always negative. Since a is necessarily positive there is only one critical value at which the upper branch of solutions loses stability:

$$a_p = 1 - \frac{1}{8b^2}\big(p + 1 - \sqrt{(p+1)^2 - 16b^2 p}\big). \tag{38}$$

Solutions of the form given in Eq. (30) are therefore stable for $0 \leq a < a_p$, and unstable for $a \geq a_p$.

Since the point a_p at which a steady-state solution depends upon the number of active genes p, Eq. (38) means that there is a series of bifurcation points $\{a_i\}$ in which different cellular configurations lose stability at different values of the bifurcation parameter a. Furthermore, the series $\{a_i\}$ is monotonic decreasing in p: that is $a_N < a_{N-1} < a_{N-2} \ldots$. So for $a < a_p$ all steady-state solutions of the form given in Eq. (30) with less than p genes active are *simultaneously* stable. That is, the system exhibits multistability. However, as a is increased above a_p the solutions with p genes active lose stability, but all others with less than p genes active remain stable. Since the sequence $\{a_i\}$ is monotonic decreasing in p, the system given by Eqs. (24) does not possess hysteresis.

Now consider transcriptional control of differentiation along the mesenchymal lineages by *RUNX2*, *SOX9* and *PPAR*-γ. In this case, there are three dimensions ($N = 3$) and the only stable nontrivial steady-state solutions are:

$$(\alpha_1, 0, 0), (0, \alpha_1, 0), (0, 0, \alpha_1); (\alpha_2, \alpha_2, 0), (\alpha_2, 0, \alpha_2), (0, \alpha_2, \alpha_2); (\alpha_3, \alpha_3, \alpha_3)$$

where $\alpha_1, \alpha_2, \alpha_3$ are determined by Eq. (32).

Notice that these solutions come in 'groups': 3 solutions have only one active gene ($p = 1$); 3 solutions have 2 active genes ($p = 2$), and one solution has all three genes active ($p = 3$). This grouping of equilibria results from the symmetry of Eqs. (24). These 'groups' of equilibria are formally known as *orbits*. In this system, the orbit of a fixed-point solution $\mathbf{x}_p^* \in \mathbb{R}^N$ is the set

$$\Delta(\mathbf{x}_p^*) = \{\gamma \mathbf{x}_p^* : \gamma \in S_N\}. \tag{39}$$

Thus, fixed-point solutions to Eqs. (24) come in S_N-orbits. The symmetry structure of Eqs. (24) ensures that all fixed-point solutions in the same orbit have the same stability. It is therefore convenient to consider orbits of steady-state solutions rather than individual solutions *per se*. We say that the orbit $\Delta(\mathbf{x}_p^*)$ is stable when $\mathbf{x}_p^*$ is stable. In the three dimensional case, the system given by Eqs. (24) has 3 distinct orbits of steady-state solutions:

$$\Delta_1 = \{(\alpha_1, 0, 0), (0, \alpha_1, 0), (0, 0, \alpha_1)\}, \tag{40}$$

$$\Delta_2 = \{(\alpha_2, \alpha_2, 0), (\alpha_2, 0, \alpha_2), (0, \alpha_2, \alpha_2)\}, \tag{41}$$

$$\Delta_3 = \{(\alpha_3, \alpha_3, \alpha_3)\}. \tag{42}$$

The first orbit represents the set of steady-state solutions in which one gene is active; the second orbit, the set of steady-state solutions in which 2 genes are active; and the third orbit the steady-state solution in which all three genes are active. Notice that these orbits order the steady-state solutions into a well-defined hierarchy: Δ_i is the set of solutions in which i genes are active.

Consider a cell in which all N master-genes are concurrently homogeneously expressed (that is, a steady-state solution in Δ_N). This configuration is stable as long as $0 \leq a < a_N$. However, as a is increased to a_N, this all-ON configuration loses stability and the cell is forced to adopt an alternative genetic state. Extensive numerical simulations suggest that this system does not admit any other more exotic solutions such as limit cycles, or chaotic trajectories. Thus, the cell has to adopt an alternative steady-state genetic configuration. S_N-equivariant bifurcation theory suggests that heterogeneous configurations do not bifurcate continuously from this homogeneous state, but rather that the system exhibits a 'jump' bifurcation, and transitions rapidly to a disparate configuration [33, 34]. Since $a_i < a_{i-1}$ for all $3 \leq i \leq N$, at the bifurcation point $a = a_N$, all orbits with less than N genes active are still stable. Thus, at bifurcation the system jumps to a configuration in an orbit with at most $N - 1$ genes active, and the cell is forced to move down a level in the orbit hierarchy. Heteroclinic connections between steady-state solutions in different orbits may be thought of as lineage-restricting differentiation events. Numerical simulations suggest that at the bifurcation point, the transition from one orbit to the next occurs rapidly and heteroclinic connections to all lower orbits exist. However we find that generically for a cell in orbit Δ_i a connection from Δ_i to Δ_{i-1} is established and the cell moves only one step down the orbit hierarchy (applying a perturbation which respects an appropriate subgroup of S_N can induce a connection from Δ_i to Δ_{i-j} for all $1 < j \leq i - 1$, however we view this as a degenerate situation). Thus, generically, at $a = a_N$ the cell moves from a steady-state in which N genes are active to a steady-state in which $N - 1$ genes are active and the last gene is switched OFF. For $a < a_{N-1}$ this state is stable, however as a is increased further to a_{N-1} this state too loses stability and the cell again moves to a lower point in orbit hierarchy exactly as before. Since the sequence of bifurcation points is monotonic decreasing, movement back up the orbit hierarchy (that is, bifurcation from Δ_i to Δ_j for $j > i$) is not possible. Thus, deterministic bifurcation through the orbit hierarchy is rigid and one-way.

We may view the orbit hierarchy in phase-space as corresponding to a well-defined biological hierarchy as follows. While cell types may be associated with attractors of genetic regulatory systems [35], stable orbits of steady-states naturally associate with *stages* of differentiation. As an example, consider differentiation along the mesenchymal lineages under the control of *RUNX2*, *SOX9* and *PPAR*-γ. When Δ_3 is stable it represents stable genetic configurations in which all 3 transcription factors are active and bifurcation to orbits Δ_2 or Δ_1 is possible. Thus, this orbit may be associated with a tripotent tissue-specific stem cell. Similarly, as a is increased bifurcation from Δ_2 to Δ_1 is possible (but not back to Δ_3) and stable steady-states in Δ_2 represent progenitor cells with bipotent differentiation potential. No bifurcation from Δ_1 back up the hierarchy is possible as a is increased, and stable solutions in Δ_1 therefore represent terminally differentiated cells in which only one master gene is active.

In the context of equivariant bifurcation theory, the process by which a system moves from a more to a less symmetric state is known as spontaneous symmetry breaking [34]. Therefore, we interpret cellular differentiation from stem- through progenitor- to differentiated cell-types to occur through a cascade of spontaneous symmetry-breaking bifurcations.

References

[1] G. Karsenty. Minireview: transcriptional control of osteoblast differentiation. *Endocrinology*, 142(7):2731–2733, 2001.

[2] T. M. Schroeder, E. D. Jensen, and J. J. Westendorf. Runx2: A master organizer of gene transcription in developing and maturing osteoblasts. *Birth Defects Res. C*, 75(3):213–225, 2005.

[3] P. Ducy, R. Zhang, V. Geoffroy, A. L. Ridall, and G. Karsenty. Osf2/Cbfa1: a transcriptional activator of osteoblast differentiation. *Cell*, 89:747–754, 1997.

[4] W. Bi, J. M. Deng, Z. Zhang, R. R. Behringer, and B. de Crombrugghe. Sox9 is required for cartilage formation. *Nat. Genetics*, 22:85–89, 1999.

[5] B. M. Spiegelman and J. S. Flier. Adipogenesis and obesity: rounding out the big picture. *Cell*, 87(3):377–389, 1996.

[6] P. Tontonoz, E. Hu, and B. M. Spiegelman. Stimulation of adipogenesis in fibroblasts by PPARγ2, a lipid-activated transcription factor. *Cell*, 79:1147–1156, 1994.

[7] O. A. MacDougald and M. D. Lane. Adipocyte differentiation: When precursors are also regulators. *Curr Biol*, 5(6):618–21, 1995.

[8] X. Wang, H. Hisha, S. Taketani, Y. Adachi, Q. Li, W. Cui, Y. Cui, J. Wang, C. Song, T. Mizokami, S. Okazakia, Q. Lia, T. Fana, H. Fane, Z. Lianf, M. E. Gershwinf, and S. Ikehara. Characterization of mesenchymal stem cells isolated from mouse fetal bone marrow. *Stem Cells*, 24(3):482–493, 2006.

[9] G. Zhou, Q. Zheng, F. Engin, E. Munivez, Y. Chen, E. Sebald, D. Krakow, and B. Lee. Dominance of SOX-9 function over RUNX-2 during skeletogenesis. *Proc. Natl. Acad. Sci.*, 103(50):19004–19009, 2006.

[10] J. H. Hong, E. S. Hwang, M. T. McManus, A. Amsterdam, Y. Tian, R. Kalmukova, E. Mueller, T. Benjamin, B. M. Spiegelman, P. A. Sharp, N. Hopkins, and M. B. Yaffe. TAZ, a transcriptional modulator of mesenchymal stem cell differentiation. *Science*, 309:1074–1078, 2005.

[11] J. H. Hong and M. B. Yaffe. TAZ: A beta-catenin-like molecule that regulates mesenchymal stem cell differentiation. *Cell Cycle*, 5:176–179, 2006.

[12] S. Yang, D. Wei, D. Wang, M. Phimphilai, P.H. Krebsbach, and RT Franceschi. In vitro and in vivo synergistic interactions between the Runx2/Cbfa1 transcription factor and bone morphogenetic protein-2 in stimulating osteoblast differentiation. *J. Bone Miner. Res.*, 18(4):705–715, 2003.

[13] B. Lecka-Czernik, E.J. Moerman, D.F. Grant, J.M. Lehmann, S.C. Manolagas, and R.L. Jilka. Divergent effects of selective peroxisome proliferator-activated receptor-γ2 ligands on adipocyte versus osteoblast differentiation. *Endocronology*, 143(6):2376–2384, 2002.

[14] E. J. Moerman, K. Teng, D. A. Lipschitz, and B. Lecka-Czernik. Aging activates adipogenic and suppresses osteogenic programs in mesenchymal marrow stroma/stem cells: the role of PPAR-gamma 2 transcription factor and TGF-beta/BMP signaling pathways. *Aging Cell*, 3:379–389, 2004.

[15] M. J. Jeon, J. A. Kim, S. H. Kwon, S. W. Kim, K. S. Park, S. W. Park, S. Y. Kim, and C. S. Shin. Activation of peroxisome proliferator-activated receptor-γ inhibits the Runx2-mediated transcription of osteocalcin in osteoblasts. *J. of Biol. Chem.*, 278(26):23270–23277, 2003.

[16] E. Khan and Y. Abu-Amer. Activation of peroxisome proliferator-activated receptor-gamma inhibits differentiation of preosteoblasts. *J. Lab. Clin. Med.*, 142(1):29–34, 2003.

[17] E. D. Rosen, C. H. Hsu, X. Wang, S. Sakai, M. W. Freeman, F. J. Gonzalez, and B. M. Spiegelman. C/EBPα induces adipogenesis through PPARγ: a unified pathway. *Genes & Development*, 16(1):22–26, 2002.

[18] B. F. Eames, P. T. Sharpe, and J. A. Helms. Hierarchy revealed in the specification of three skeletal fates by Sox-9 and Runx-2. *Dev. Biol.*, 274(1):188–200, 2004.

[19] S. Harada and G. A. Rodan. Control of osteoblast function and regulation of bone mass. *Nature*, 423:349–355, 2003.

[20] K. Nakashima, X. Zhou, G. Kunkel, Z. Zhang, J. M. Deng, R. R. Behringer, and B. de Crombrugghe. The novel zinc finger-containing transcription factor osterix is required for osteoblast differentiation and bone formation. *Cell*, 108(1):17–29, 2002.

[21] F. Legendre, J. Dudhia, J. P. Pujol, and P. Bogdanowicz. JAK/STAT but Not ERK1/ERK2 pathway mediates interleukin (IL)-6/Soluble IL-6R down-regulation of type II collagen, aggrecan core, and link protein transcription in articular chondrocytes. *J. Biol. Chem.*, 278(5):2903–2912, 2003.

[22] J. M. Stephens, R. F. Morrison, Z. Wu, and S. R. Farmer. PPAR-γ Ligand-dependent induction of STAT1, STAT5A, and STAT5B during adipogenesis. *Biochem. Biophys. Res. Commun.*, 262(1):216–222, 1999.

[23] T. Furumatsu, M. Tsuda, N. Taniguchi, Y. Tajima, and H. Asahara. Smad3 induces chondrogenesis through the activation of SOX9 via CREB-binding protein/p300 recruitment. *J. Biol. Chem.*, 280(9):8343–8350, 2005.

[24] L. Choy and R. Derynck. Transforming growth factor-β inhibits adipocyte differentiation by Smad3 interacting with CCAAT/enhancer-binding protein (C/EBP) and repressing C/EBP transactivation function. *J. Biol. Chem.*, 278(11):9609–9619, 2003.

[25] H. Drissi, Q. Luc, R. Shakoori, S. Chuva De Sousa Lopes, J.Y. Choi, A. Terry, M. Hu, S. Jones, J.C. Neil, J.B. Lian, J. L. Stein, A. J. van Wijnen, and G. S. Stein. Transcriptional autoregulation of the bone related CBFA-1/RUNX-2 gene. *J. Cell. Physiol.*, 184:341–350, 2000.

[26] I. Roeder and I. Glauche. Towards an understanding of lineage specification in hematopoietic stem cells: a mathematical model for the interaction of transcription factors GATA-1 and PU. 1. *J. Theor. Biol.*, 241:852–865, 2006.

[27] C. Duan and D. R. Clemmons. Transcription factor AP-2 regulates human insulin-like growth factor binding protein-5 gene expression. *J. Biol. Chem.*, 270(42):24844–24851, 1995.

[28] W. F. Xie, S. Kondo, and L. J. Sandell. Regulation of the mouse cartilage-derived retinoic acid-sensitive protein gene by the transcription factor AP-2. *J. Biol. Chem.*, 273(9):5026–5032, 1998.

[29] P. Kannan, R. Buettner, P. J. Chiao, S. O. Yim, M. Sarkiss, and M. A. Tainsky. N-ras oncogene causes AP-2 transcriptional self-interference, which leads to transformation. *Genes and Development*, 8(11):1258–1269, 1994.

[30] J. D. Murray. *Mathematical biology: I. an introduction.* Springer-Verlag, New York, 2002.

[31] J. Kawaguchi, P. J. Mee, and A. G. Smith. Osteogenic and chondrogenic differentiation of embryonic stem cells in response to specific growth factors. *Bone*, 36:758–769, 2005.

[32] N. Ivanova, R. Dobrin, R. Lu, I. Kotenko, J. Levorse, C. DeCoste, X. Schafer, Y. Lun, and I. R. Lemischka. Dissecting self-renewal in stem cells with RNA interference. *Nature*, 442:533–538, 2006.

[33] T. Elmhirst. S_N-equivariant symmetry-breaking bifurcations. *Int. J. Bifurcat. Chaos.*, 14:1017–1036, 2004.

[34] M. Golubitsky and I. Stewart. *The symmetry perspective.* Birkhäuser Verlag, Basel, 2002.

[35] S. A. Kauffman. *The origins of order: self-organization and selection in evolution.* Oxford University Press, Oxford, 1993.
